# Participation of the PI-3K/Akt-NF-κB signaling pathways in hypoxia-induced mitogenic factor-stimulated Flk-1 expression in endothelial cells

**DOI:** 10.1186/1465-9921-7-101

**Published:** 2006-07-27

**Authors:** Qiangsong Tong, Liduan Zheng, Li Lin, Bo Li, Danming Wang, Chuanshu Huang, George M Matuschak, Dechun Li

**Affiliations:** 1 Department of Internal Medicine, Saint Louis University, Saint Louis, MO 63110, USA; 2 Department of Pathology, Union Hospital of Tongji Medical College, Huazhong University of Science and Technology, Wuhan, Hubei 430022, China; 3 Department of Medicine, Johns Hopkins University School of Medicine, Baltimore, MD 21287, USA; 4 Nelson Institute of Environmental Medicine, New York University School of Medicine, Tuxedo, NY 10987, USA

## Abstract

**Background:**

Hypoxia-induced mitogenic factor (HIMF), a lung-specific growth factor, promotes vascular tubule formation in a matrigel plug model. We initially found that HIMF enhances vascular endothelial growth factor (VEGF) expression in lung epithelial cells. In present work, we tested whether HIMF modulates expression of fetal liver kinase-1 (Flk-1) in endothelial cells, and dissected the possible signaling pathways that link HIMF to Flk-1 upregulation.

**Methods:**

Recombinant HIMF protein was intratracheally instilled into adult mouse lungs, Flk-1 expression was examined by immunohistochemistry and Western blot. The promoter-luciferase reporter assay and real-time RT-PCR were performed to examine the effects of HIMF on Flk-1 expression in mouse endothelial cell line SVEC 4–10. The activation of NF-kappa B (NF-κB) and phosphorylation of Akt, IKK, and IκBα were examined by luciferase assay and Western blot, respectively.

**Results:**

Intratracheal instillation of HIMF protein resulted in a significant increase of Flk-1 production in lung tissues. Stimulation of SVEC 4–10 cells by HIMF resulted in increased phosphorylation of IKK and IκBα, leading to activation of NF-κB. Blocking NF-κB signaling pathway by dominant-negative mutants of IKK and IκBα suppressed HIMF-induced Flk-1 upregulation. Mutation or deletion of NF-κB binding site within Flk-1 promoter also abolished HIMF-induced Flk-1 expression in SVEC 4–10 cells. Furthermore, HIMF strongly induced phosphorylation of Akt. A dominant-negative mutant of PI-3K, Δp85, as well as PI-3K inhibitor LY294002, blocked HIMF-induced NF-κB activation and attenuated Flk-1 production.

**Conclusion:**

These results suggest that HIMF upregulates Flk-1 expression in endothelial cells in a PI-3K/Akt-NF-κB signaling pathway-dependent manner, and may play critical roles in pulmonary angiogenesis.

## Background

Vascular endothelial growth factor (VEGF) is essential for many angiogenic processes in both normal and pathological conditions [1,2]. The biological activities of VEGF are mediated mainly through two tyrosine kinase receptors, fms-like tyrosine kinase-1(Flt-1) and fetal liver kinase-1/kinase-insert domain receptor (Flk-1/KDR), whose expressions are mainly restricted to endothelial cells [1,2]. These receptors are membrane-spanning receptor tyrosine kinases that bind VEGF with high affinity. Flk-1 is now considered to be the main receptor involved in endothelial cell proliferation, migration, survival, and the dominant form in pulmonary vascular system [2,3]. In contrast, Flt-1 has a decoy effect on VEGF signaling, possibly with variations related to the vascular bed type [2]. Both Flt-1- and Flk-1-deficient mice die *in utero *between embryonic days (E) 8.5 and E 9.5 but have different phenotypes. Flt-1-deficient embryos showed an overgrowth of endothelial cells, disorganization of blood vessels [4], and normal vascular development [5], suggesting that the Flt-1 tyrosine kinase is not necessary for vasculogenesis during development. On the other hand, Flk-1-deficient mice lack both mature endothelial and hematopoietic cells, indicating that Flk-1 is crucial for vascular development of both endothelial and hematopoietic precursors [6]. During later stages of embryonic development, Flk-1 is highly expressed on endothelial cells, but is down-regulated in most hematopoietic cells [7]. In the adult, the expression level of Flk-1 is low, restricted to endothelial cells and transiently upregulated during angiogenesis [8].

*In vitro *studies have shown that Flk-1 expression is temporally regulated by several growth factors [2] and by shear stress [9]. For example, both basic fibroblast growth factor (bFGF) and tumor necrosis factor-α(TNF-α) have been shown to induce expression of the endogenous Flk-1 gene and increase Flk-1 upstream promoter activity in cultured endothelial cells [10,11]. It has been known that shear stress induces Flk-1 expression through the CT-rich Sp1 binding site within Flk-1 promoter [9]. Incubation of cells with the multifunctional angiogenic cytokine transforming growth factor β1 (TGF-β1) results in a rapid and marked decrease in Flk-1 expression levels and cell surface ^125^I-VEGF binding capacity [12]. Because expression of Flk-1 is highly restricted to endothelial cells and tightly controlled during angiogenesis, further understanding of the potential factors that regulate the expression of Flk-1 in the lung and endothelium would provide general insights into the mechanisms of vascular development in health and diseases in the pulmonary circulation.

Hypoxia-induced mitogenic factor (HIMF) is a secreted protein from airway epithelial cells and alveolar type II cells and it is originally discovered in a mouse model of hypoxia-induced pulmonary hypertension [13]. Subsequent studies showed that HIMF is a lung-specific growth factor participating in lung cell proliferation and modulation of compensatory lung growth [13,14]. HIMF possesses an angiogenic function that promotes vascular tubule formation in a matrigel plug model [13], and is developmentally regulated and exhibits antiapoptotic functions [15]. Moreover, our recent studies have indicated that HIMF modulates surfactant protein B and C expression in lung epithelial cells [16]. We have also established that HIMF promotes VEGF production in alveolar type II cells, indicating HIMF may play critical roles in angiogenesis in the pulmonary system [17]. In this study, we further investigated the molecular mechanisms of HIMF on Flk-1 expression in mouse lungs, and in cultured endothelial cells. The results showed that HIMF promotes expression of Flk-1 via activation of PI-3 kinase/Akt and NF-κB signaling pathways.

## Materials and methods

### Animal experiments

Adult male C57Bl/6 mice (10–12 weeks old) were obtained from Jackson Laboratories (Bar Harbor, ME). Recombinant HIMF protein was produced in TREx 293 cells and purified as previously described [13]. Intratracheal instillation of HIMF protein or bovine serum albumin (BSA, Sigma, St. Louis, MO) were performed as previously reported [14,16]. All experiments followed the protocols approved by the Animal Care and Use Committee of Saint Louis University.

### Immunohistochemical and immunofluorescent staining for Flk-1

Lung samples were processed and immunostained as previously described [13,15,16]. Briefly, the sections were incubated for 1 hour with anti-Flk-1 antibody (Santa Cruz Biotechnology, Inc., Santa Cruz, CA; 1:200 dilution) followed by a 2-hour incubation with goat anti-rabbit antibodies conjugated with HRP or FITC (1: 400 dilution, Bio-Rad, Hercules, CA). For immunofluorescent staining, the cells were examined directly under a fluorescent microscope after secondary antibody incubation and washing. For immunohistochemical staining, DAB substrate (Dako, Carpinteria, CA) was used to generate dark brown precipitate in the cells of the tissues. The images were taken with a Sony color digital DXC-S500 camera (Sony Electronics, Oradell, NJ), using Image Pro-Express software (Media Cybernetics, Silver Spring, MD).

### Western blot for HIMF, Flk-1, VEGF, and GAPDH

Tissue collection, homogenization, and protein electrophoresis were performed as previously described [14-16]. Protein (50 μg) or 40 μl of medium supernatant (for HIMF expression assay in cultured cells) from each sample was subjected to 4–20% pre-cast polyacrylamide gel electrophoresis (Bio-Rad, Hercules, CA). HIMF, Flk-1, VEGF, and GAPDH were detected with 1:1000, 1:500, 1:500 and 1:1000 dilutions of antibodies, respectively, followed by 1:3000 dilution of goat anti-rabbit HRP-labeled antibody (Bio-Rad). ECL substrate kit (Amersham, Piscataway, NJ) was used for the chemiluminscent detection of the signals with autoradiography film (Amersham).

### Real-time RT-PCR for HIMF, Flk-1, and VEGF

Total RNA was isolated with RNeasy Mini Kit (Qiagen Inc., Valencia, CA). The reverse transcription reactions were conducted with Transcriptor First Strand cDNA Synthesis Kit (Roche, Indianapolis, IN). Real-time PCR with SYBR Green PCR Master Mix (Applied Biosystems, Foster City, CA) was performed using ABI Prism 7700 Sequence Detector (Applied Biosystems). The PCR primers were the following: for mouse HIMF 5'-ATGAA GACTACAACTTGTTCCC-3' (positions 104 to 125 of second exon) and 5'-TTAGGACAGT TGGCAGCAGCG-3' (positions 419 to 439 of fourth exon) amplifying a 336-bp fragment; for mouse Flk-1 5'-GCATCACCAGCAGCCAGAG-3' and 5'-GGGCCATCCACTTCAAAGG-3' amplifying a 327-bp fragment between positions 3095 and 3421; for mouse VEGF 5'-TGGAT GTCTACCAGCGAAGC-3' and 5'-ACAAGGCTCACAGTGATTTT-3' amplifying a 308-bp fragment between positions 522 and 829; for mouse GAPDH, 5'-GCCAAGGTCATCCATGA CAACTTTGG-3' and 5'-GCCTGCTTCACCACCTTCTTGATGTC-3' amplifying a 314-bp fragment between positions 532 and 845.

### Cell culture and stimulation with HIMF

SVEC 4–10, an SV40-transformed murine endothelial cell line [18], was obtained from the ATCC (CRL-2181) and grown in Dulbecco's Minimal Eagles Medium (DMEM, Gibco Laboratories, Grand Island, NY) supplemented with 10% fetal bovine serum (FBS, Gibco), penicillin (100 U/ml) and streptomycin (100 μg/ml). Cells were maintained at 37°C in a humidified atmosphere of 5% CO_2_. After the cells reached 80–90% confluency, the cells were fed with a medium supplemented with 0.1% FBS and 2 mmol/L L-glutamine. Thirty-three hours later, cells were incubated in serum-free DMEM for 4 h, and pretreated with LY294002, SB203580, PD98059 or U0126 (Calbiochem, La Jolla, CA) as indicated, then stimulated with different concentrations of HIMF protein for specified periods, with or without Actinomycin D (5 μg/ml, Sigma).

### Transfection and stable cell lines

HIMF cDNA vector, dominant-negative mutants of IKKα [IKKα (K44A)], IKKβ [IKKβ (K44A)], IκBα super-repressor [IκBα (S32A/S36A)] and phosphatidylinositol 3-kinase (PI-3K) dominant negative mutant (Δp85) were previously described [16,19,20]. HIMF cDNA or dominant-negative mutants were transfected into SVEC 4–10 cells with Lipofectamine 2000 (Life Technologies, Inc., Gaithersburg, MD). Stable cell lines, SVEC-HIMF, and their transfection control (vector only) cells SVEC-Zeo, were selected with Zeocin (400 μg/ml). HIMF expression was validated by Western blot and real-time RT-PCR analyses.

### Dual-luciferase reporter assay for Flk-1 and NF-κB

Mouse Flk-1 5'-flanking regions (-258/+299, -96/+299, -71/+299, and -36/+299 bp; GenBank accession No. AF153057) were amplified by PCR from genomic DNA obtained from SVEC 4–10 and subcloned into the *Kpn*I-*Hind*III site of pGL3-Basic (Invitrogen, Carlsbad, CA), a *firefly *luciferase reporter vector. Mutagenesis and deletion of NF-κB binding site within Flk-1 promoter were performed using the GeneTailor Site-Directed Mutagenesis System (Invitrogen). Mutation and deletion oligonucleotides for NF-κB binding site were designed as follows: forward mutation 5'-TATCGATAGGTACCGGACGCACCGAGTCCCCACCCCT, forward deletion 5'-TATCGATAGGTACCGGACGCACCCCACCCCT, reverse 5'-TGCGTC CGGTACCTATCGATAGAG AAATGTT. The DNA constructs were verified by sequence analysis. The NF-κB *firefly *luciferase reporter vector, pNFκB-Luc (Stratagene, La Jolla, CA), is designed to measure the binding of transcription factors to the κ enhancer. It contains five tandem repeats of NF-κB binding sites (TGGGGACTTTCCGC) as promoters upstream of the luciferase transcription start site in the vector. The expression of luciferase gene in the reporter plasmid is controlled by these NF-κB binding sequences. Only when there is activated NF-κB in the nucleus (translocated NF-κB), the luciferase transcription and translation start. By measuring the luciferase activity in the transfected cell lysats, it provides an indirect evidence of NF-κB activation in the nucleus. Cells were co-transfected with each reporter construct and the *renilla *luciferase vector pRL-TK (Promega, Madison, WI), with or without HIMF protein stimulation, and then treated with passive lysis buffer according to the dual-luciferase assay manual (Promega). The luciferase activity was measured with a luminometer (Lumat LB9507, Berthold Tech., Bad Wildbad, Germany). The *firefly *luciferase signal was normalized to the *renilla *luciferase signal for each individual analysis to eliminate the variations of transfection efficiencies.

### Phosphorylation assay for IKK, IκBα, Akt, and MAPK

SVEC 4–10 cells were treated with HIMF as described above. Protein (50 μg) from each sample was subjected to 4–20% pre-cast polyacrylamide gel (Bio-Rad) electrophoresis and transferred to nitrocellulose membranes (Bio-Rad), and then probed with rabbit anti-mouse antibodies against phospho-specific and non-phosphorylated IKK, IκBα, Akt, ERK1/2, p38 kinase, and JNK mitogen-activated protein kinase (MAPK) (1:500 dilutions, Santa Cruz Biotechnology), followed by 1:3000 dilution of goat anti-rabbit HRP-labeled antibody (Bio-Rad). ECL substrate kit (Amersham) was used for the chemiluminscent detection of the signals with autoradiography film (Amersham).

### Statistical analysis

Unless otherwise stated, all data were shown as mean ± standard error of the mean (SEM). Statistical significance (*P *< 0.05) was determined by *t *test or analysis of variance (ANOVA) followed by assessment of differences using SigmaStat 2.03 software (Jandel, Erkrath, Germany).

## Results

### HIMF enhances Flk-1 expression in mouse lung tissues

**Figure 1:**
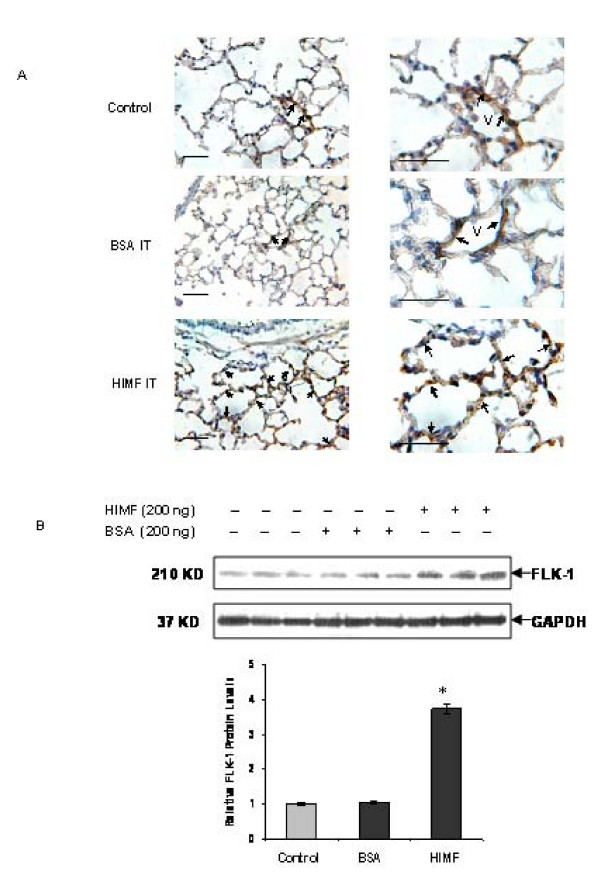
**HIMF enhances Flk-1 expression in mouse lungs**. Recombinant HIMF protein or BSA was intratracheally instilled into adult mice (200 ng/animal in 40 μl saline, n = 3 for each group). The vehicle controls were instilled with saline (40 μl/animal, n = 3). Twenty-four hours later, the mouse lungs were collected. (A) Immunohistochemical staining results indicated that instillation of HIMF protein, but not BSA, resulted in a significant increase of Flk-1 production, mainly located at endothelial cells of the alveolar capillaries (arrows). However, the Flk-1 staining is very weak in the alveolar septa and strong signal is only found in vascular endothelial cells (v) in both saline and BSA controls (arrows). Scale bars: 100 μm. (B) Western blot with proteins from lung homogenates indicated that Flk-1 expression was enhanced in HIMF-, but not in saline- or BSA-instilled mouse lungs. The symbol (*) indicates a significant increase from control mouse lungs instilled with saline only (*P *< 0.05).

To examine the role of HIMF in Flk-1 expression, we intratracheally instilled recombinant HIMF protein into adult mouse lungs. We found that Flk-1 expression was significantly enhanced by HIMF stimulation, as demonstrated by positive immunohistochemical staining mainly located in alveolar capillary endothelial cells (Fig 1A). In contrast, low level of Flk-1 expression was only observed in endothelial cells of small pulmonary vessels and very rarely seen in the capillary endothelial cells of alveolar walls in the control mouse lungs treated with either saline or BSA (Fig 1A). Western blotting further confirmed the upregulation of Flk-1 in lung tissues after 24 h of HIMF-instillation, but not in the saline or BSA control lungs (Fig 1B).

### HIMF upregulates Flk-1, but not VEGF, expression in mouse endothelial cells

**Figure 2:**
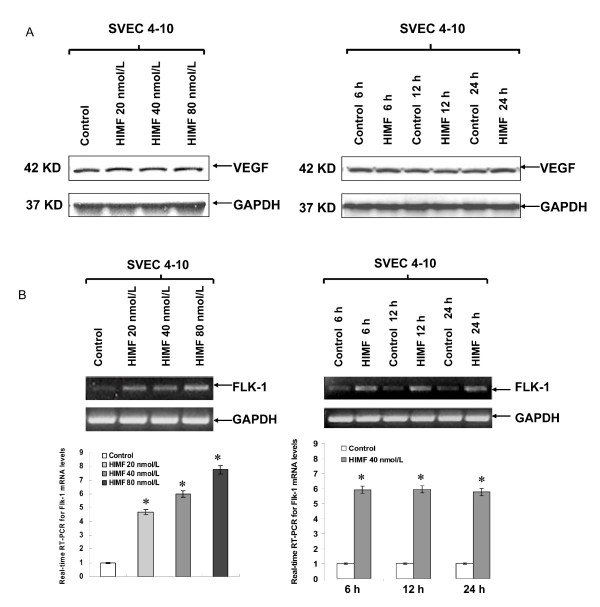
**HIMF induces Flk-1, but not VEGF, expression in mouse endothelial cell line**. Endothelial SVEC 4–10 cells were treated with HIMF for various concentrations and periods as indicated. Western blot for VEGF and real-time RT-PCR for Flk-1 expression were performed. (2A) HIMF administration had no impact on VEGF expression in SVEC 4–10 cells. (2B) HIMF induced Flk-1 transcript increase in SVEC 4–10 cells in a dose-dependent manner. Time-course study indicated that HIMF (40 nmol/L)-induced Flk-1 expression can be detected at 6 h, and persisted for 24 h. Triplicate experiments were performed with essentially identical results (n = 3).

**Figure 3:**
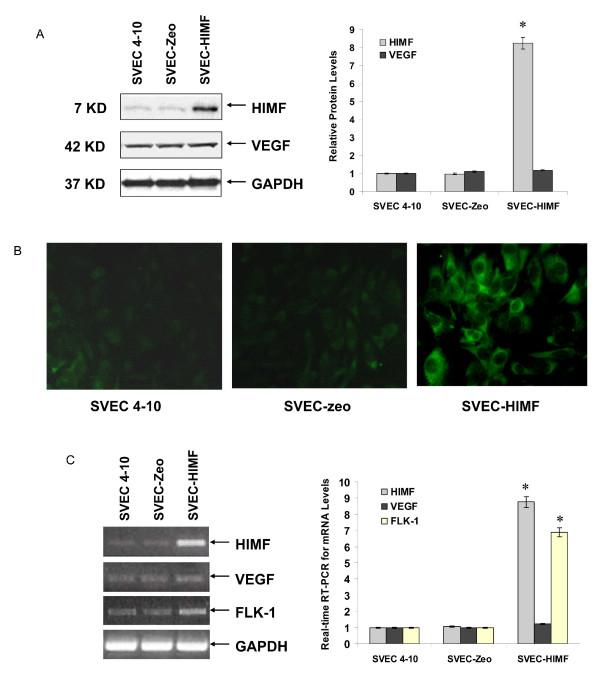
**Generation of HIMF overexpressing endothelial cells**. SVEC 4–10 cells were transfected with HIMF cDNA or control vector. Stable cell lines, SVEC-HIMF, along with their transfection control cells SVEC-Zeo, were screened based on resistance to Zeocin (400 μg/ml). Western blots with cell culture medium for HIMF and protein from cell lysate for VEGF (3A), immunofluorescence staining for Flk-1 (3B) and real-time RT-PCR with cell total RNA (3C) demonstrated that SVEC-HIMF cells have higher HIMF protein and mRNA levels than their parent (SVEC 4–10) and vector-transfection (SVEC-Zeo) counterparts. The levels of Flk-1, but not VEGF, in SVEC-HIMF were also increased significantly compared with those of their controls. The symbol (*) indicates a significant increase from parent controls (*P *< 0.05). Triplicate experiments were performed with essentially identical results (n = 3).

Although HIMF treatment leads to upregulation of Flk-1, molecular mechanisms governing such induced expression in lung tissues remain unclear. To establish a cellular system for further investigating regulatory mechanisms of HIMF-induced Flk-1 production, we used cultured endothelial SVEC 4–10 cells as models [18]. Western blotting of cell lysates and real-time RT-PCR with cell total RNA showed that HIMF induced Flk-1, but not VEGF production, in a dose-dependent manner in SVEC 4–10 cells (Fig. 2A and 2B). Time-course studies showed that HIMF-induced Flk-1 expression was detectable at 6 h, and sustained for 24 h (Fig. 2B). Flk-1, but not VEGF, protein and mRNA were also expressed in an elevated level in a cell line, SVEC-HIMF that stably expresses HIMF (Fig. 3A, 3B and 3C). Successful recapitulation of HIMF-induced Flk-1 expression in endothelial cell line provided the basis for further dissecting the molecular mechanism of HIMF-induced upregulation of Flk-1.

### HIMF increases Flk-1 transcription rather than its mRNA stability

**Figure 4:**
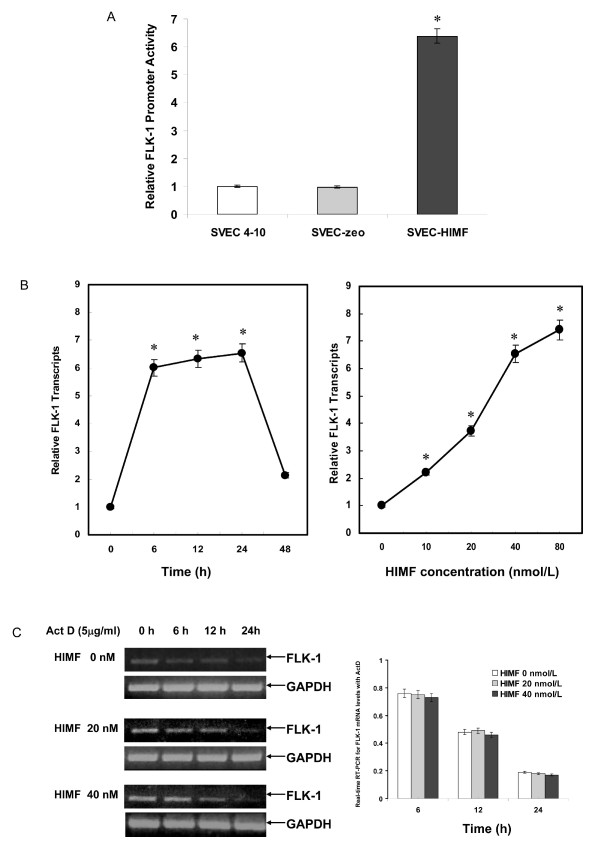
**HIMF increases the transcription activities, but not mRNA stability, of Flk-1 in SVEC 4–10 cells**. (4A) SVEC 4–10, SVEC-zeo and SVEC-HIMF cells were co-transfected with pGL-Flk-1 (-258/+299) and pRL-TK. Twenty-four hours later, cells were lysed with passive lysis buffer, and luciferase activity was measured according to the dual-luciferase assay manual. The results indicated that SVEC-HIMF cells have higher Flk-1 transcription activities than those of their controls. (4B) SVEC 4–10 cells were co-transfected with pGL-Flk-1 (-258/+299) and pRL-TK. Twenty-four hours later, the cells were incubated with HIMF protein as indicated. Then, cells were lysed with passive lysis buffer, and luciferase activity was measured according to the dual-luciferase assay manual. The time-course study demonstrated that HIMF (40 nmol/L)-induced Flk-1 transcription is detectable at 6 h, and persisted for 24 h. After incubation with 10–80 nmol/L of HIMF, Flk-1 promoter activities in SVEC 4–10 were enhanced in a dose-dependent manner. (4C) SVEC 4–10 were treated with different concentrations of HIMF and incubated with 5 μg/ml of Actinomycin D for 6, 12 and 24 h. Real-time RT-PCR indicated that HIMF did not prevent Flk-1 degradation when treated with Actinomycin D in SVEC 4–10 cells. The symbol (*) indicates a significant increase from SVEC 4–10 controls without HIMF (*P *< 0.05). Triplicate experiments were performed with essentially identical results (n = 3).

To test whether HIMF enhances Flk-1 expression at transcriptional level, we used a reporter construct, pGL-Flk-1 (-258/+299), which contains a luciferase gene driven by the Flk-1 5'-upstream proximal promoter. The reporter plasmid was transiently transfected into SVEC-HIMF, which resulted in higher Flk-1 promoter activities than those of its counterparts (Fig. 4A). HIMF treatment of pGL-Flk-1(-258/+299)-transfected SVEC 4–10 cells induced significant increases of luciferase activity in a dose-dependent manner (Fig. 4B). It has been reported that Flk-1 mRNA stability is an important posttranscriptional parameter that modulates Flk-1 expression [21]. It is, therefore, possible that HIMF treatment enhances Flk-1 mRNA stability. To test this possibility, we used Actinomycin D, a transcription inhibitor that blocks transcription. However, Flk-1 mRNA degradation was still observed when treatment of SVEC 4–10 cells with HIMF and Actinomycin D (Fig. 4C). These observations suggest that HIMF does not influence Flk-1 mRNA stability and the regulation of Flk-1 expression by HIMF is at transcriptional, rather than posttranscriptional level.

### Activation of NF-κB is essential for HIMF-induced Flk-1 expression

**Figure 5:**
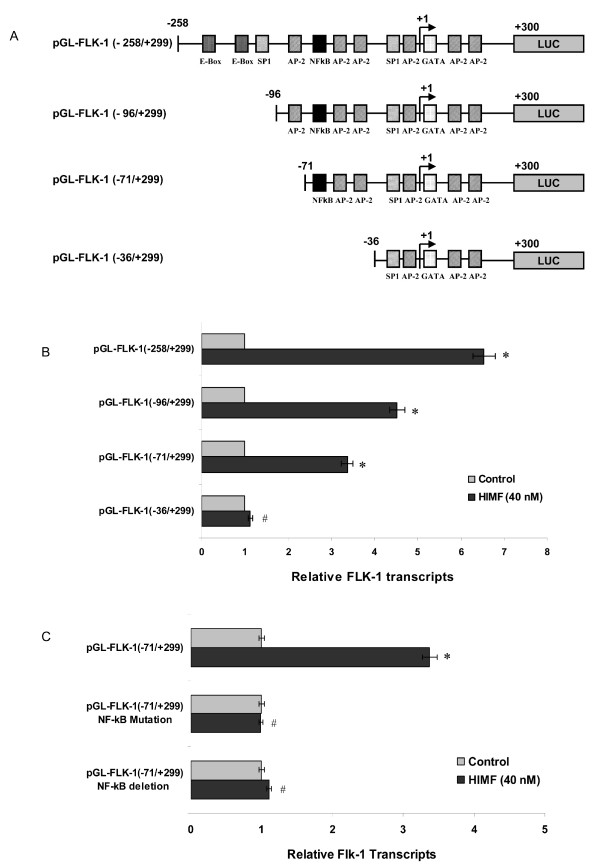
**Promoter deletion assay for HIMF-induced Flk-1 expression in SVEC 4–10 cells**. SVEC 4–10 cells were co-transfected with pRL-TK and each Flk-1 luciferase reporter construct (5A) for 24 h, then cells were incubated with HIMF protein (40 nmol/L) for another 24 h. Luciferase activity was measured and the *firefly *luciferase signal was normalized to the *renilla *luciferase signal for each individual well. (5B) HIMF induced high Flk-1 promoter activities within cells transfected with pGL-Flk-1 (-258/+299), pGL-Flk-1 (-96/+299) or pGL-Flk-1 (-71/+299), which contain one NF-κB binding site within Flk-1 promoter. Deletion of binding sites for E-Box, Sp1 and AP-2 partially attenuated the transcription activity. In addition, deletion of NF-κB binding site completely abolished HIMF-induced Flk-1 promoter activity. (5C) Further mutation or deletion NF-κB binding site within pGL-Flk-1 (-71/+299) abolished HIMF-induced Flk-1 transcripts in SVEC 4–10 cells. The symbol (*) indicates a significant increase from SVEC 4–10 controls treated without HIMF (*P *< 0.05). The symbol (#) indicates a significant decrease from SVEC 4–10 transfected with pGL-Flk-1 (-258/+299) or pGL-Flk-1 (-71/+299) and treated with HIMF (*P *< 0.05). Triplicate experiments were performed with essentially identical results (n = 3).

**Figure 6:**
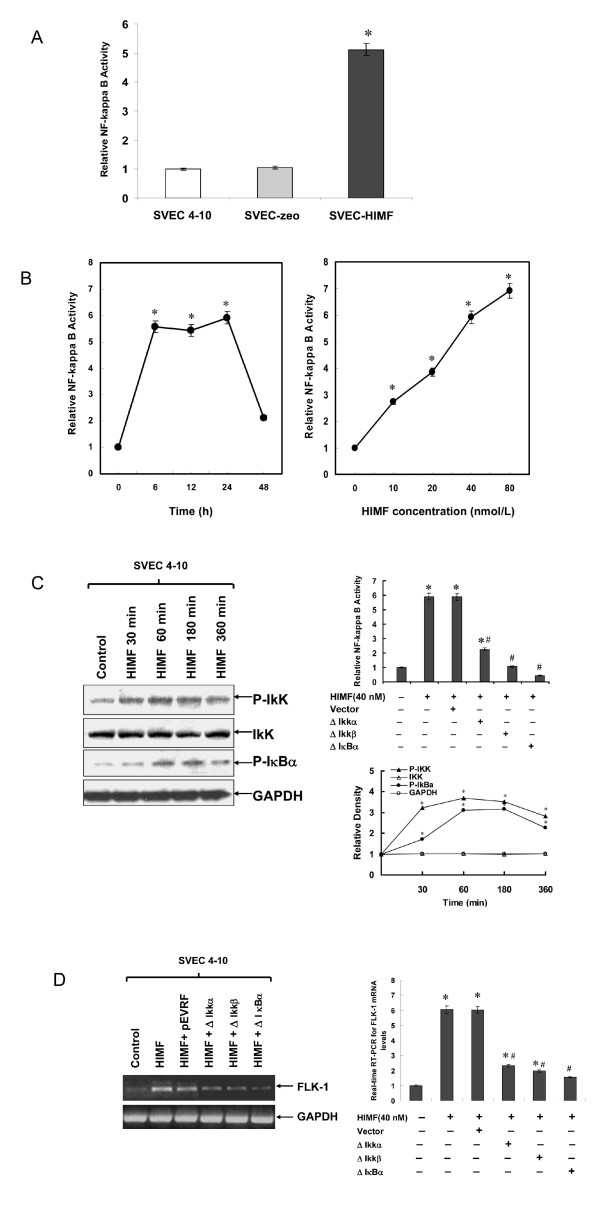
**Activation of NF-κB is essential for HIMF-induced Flk-1 expression**. Cells were co-transfected with pNFκB-luc, dominant-negative mutants of NF-κB pathway and pRL-TK, with or without stimulation of HIMF protein for various periods as indicated. (6A) Dual-luciferase assay indicated that SVEC-HIMF had higher NF-κB activity than their control counterparts. (6B) Dual-luciferase assay indicated that HIMF protein increased NF-κB activity in SVEC 4–10 cells in a dose-dependent manner. (6C) Western blots indicated that HIMF (40 nmol/L) induced phosphorylation of IKK and IκBα in SVEC 4–10 cells. Transfection of SVEC 4–10 cells with dominant-negative mutants IKKα (K44A) and IKKβ (K44A), or super-repressor IκBα (S32A/S36A) abolished HIMF (40 nmol/L)-induced NF-κB activity. The figures indicate the relative density compared to control. (6D) The upregulation of Flk-1 induced by HIMF (40 nmol/L) in SVEC 4–10 cells were also attenuated by transfection of these dominant-negative mutants. The symbol (*) indicates a significant increase from SVEC 4–10 parent controls or controls treated without HIMF (*P *< 0.05). The symbol (#) indicates a significant decrease from SVEC 4–10 cells treated with HIMF only (*P *< 0.05). Triplicate experiments were performed with essentially identical results (n = 3).

Since HIMF enhances Flk-1 expression at transcriptional level, we further explored the possible transcription factor(s) involved in Flk-1 gene expression regulation. We generated a series of luciferase reporter constructs containing different deletion segments of mouse Flk-1 promoter sequence [22], including binding sites for E-Box, Sp1, AP-2 and NF-κB (Fig. 5A). As shown in Fig. 5B and 5C, deletion binding sites for E-Box, Sp1, and AP-2 attenuated Flk-1 promoter activity by 50%, indicating these transcription factors also play important roles in Flk-1 expression. However, deletion or mutation of NF-κB binding site completely abolished HIMF-induced Flk-1 promoter activity in SVEC 4–10 cells (Fig. 5C). It has been reported that activation of NF-κB leads to the expression of Flk-1 [23]. We therefore tested whether HIMF induction would lead to activation of NF-κB, and subsequently, enhances expression of Flk-1 using luciferase reporter assays. As shown in Fig. 6A, NF-κB activities in SVEC-HIMF were significantly higher than those of their control counterparts. Consistent with the observation in SVEC-HIMF cell line, incubation of SVEC 4–10 cells with HIMF protein also induces NF-κB activity in a dose-dependent manner (Fig. 6B). The prerequisite of NF-κB activation is the signal-dependent activation of the IKK-signalsome that contains IKKα and β kinases [23]. We found that HIMF induces phosphorylation of IKK and IκBα in SVEC 4–10 cells (Fig. 6C), suggesting that HIMF signal, at least partly, mediated through NF-κB route. Transfection of dominant negative mutants of IKK kinases, IKKα (K44A) and IKKβ (K44A), and an IκBα super-repressor, I κBα (S32A/S36A), abolished HIMF-induced NF-κB activity and Flk-1 production in SVEC 4–10 cells (Fig. 6C and 6D). Together, these findings demonstrated that activation of transcription factor NF-κB is essential for HIMF-induced Flk-1 expression.

### PI-3K/Akt pathway is involved in HIMF-induced NF-κB activation and Flk-1 production

**Figure 7:**
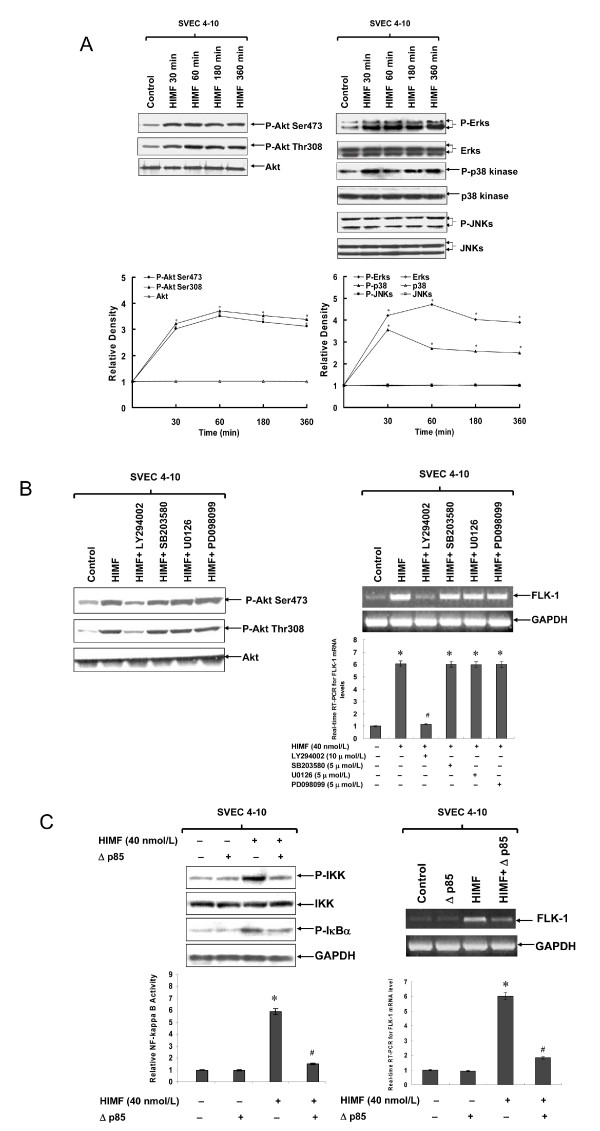
**HIMF-induced NF-κB activation and upregulation of Flk-1 are PI-3K/Akt pathway dependent**. SVEC 4–10 cells were pretreated with signal transduction inhibitors or co-transfected with luciferase constructs and PI-3K dominant-negative mutant, then stimulated with HIMF (40 nmol/L) for various periods as indicated. (7A) HIMF strongly induces phosphorylation of Akt at Ser473 and Thr308. The Akt phosphorylation is detectable at 30 minutes and sustained for 360 min. HIMF also induced phosphorylation of ERK1/2 and p38 MAPK, but not JNKs, in SVEC 4–10 cells. The figures indicate the relative density compared to control. (7B) The PI-3K inhibitor LY294002 (10 μmol/L), but not SB203580 (5 μmol/L), PD098059 (5 μmol/L) or U0126 (5 μmol/L), abolished HIMF-induced Akt phosphorylation and upregulation of Flk-1 in SVEC 4–10 cells. (7C) Transfection of Δp85 into SVEC 4–10 cells abolished HIMF-induced phosphorylation of IKK and IκBα, prevented NF-κB activation and production of Flk-1. The symbol (*) indicates a significant increase from SVEC 4–10 controls without HIMF treatment (*P *< 0.05). The symbol (#) indicates a significant decrease from SVEC 4–10 cells treated with HIMF only (*P *< 0.05). Triplicate experiments were performed with essentially identical results (n = 3).

It has been reported that HIMF activates PI-3K/Akt signaling pathway in lung epithelial cells [17]. It is unclear, though, whether there is interplay between PI-3K/Akt and NF-κB pathways in endothelial cells, and whether such interplay is necessary for HIMF-induced Flk-1 production. We therefore first tested the activation of main components of PI-3K/Akt signaling pathway upon HIMF treatment by Western blot. As shown in Fig. 7A, HIMF strongly induced phosphorylation of Akt at Ser473 and Thr308, ERK1/2, and p38 MAPK, but not JNK MAPK in SVEC 4–10 cells. The Akt activation was detectable at 30 min upon HIMF treatment, and sustained till 360 min. The PI-3K inhibitor LY294002 suppressed HIMF-induced Akt phosphorylation and upregulation of Flk-1 (Fig. 7B). Inhibitors to p38 and ERK1/2 MAPK pathways, SB203580, PD098059 or U0126, respectively, did not block Akt phosphorylation and had no effects on HIMF-induced Flk-1 expression (Fig. 7B). Further, we found that transfection of Δp85, a dominant-negative mutant of PI-3K, into SVEC 4–10 cells abolished HIMF-induced phosphorylation of IKK and IκBα (Fig. 7C), suggesting that PI-3K signaling acts at upstream of IKK signalsome. Consistent with this notion, Δp85 also blocked HIMF-induced NF-κB activation as demonstrated by reduced NF-κB luciferase activity, and the production of Flk-1 transcripts (Fig. 7C). These results strongly suggest that the interplay between PI-3K/Akt and NF-κB signaling pathways is essential for HIMF-induced Flk-1 expression in endothelial cells.

## Discussion

Endothelial cell tyrosine kinase receptors are of fundamental importance in transmission of both differentiation and angiogenic signals from the extracellular environment to the endothelium. Five endothelial cell-specific tyrosine kinase receptors, each of which has a specific role in blood vessel formation, have been identified. These include Tie-1, Tie-2 (also known as Tek), Flt-1, Flt-4, and Flk-1/KDR [24]. While the ligands for Tie-1 and Tie-2 have not yet been identified, Flk-1 and Flt-1 are receptors for VEGF [1,2], an endothelial cell-specific mitogen whose importance in both physiological and pathological angiogenesis is well established [1,2]. One of the important functions of Flk-1 is the stimulation of vascular endothelial cell survival, growth, and promotion of angiogenesis. In the lung, Flk-1 also plays central roles in alveolar formation. It is worthy to note that coordinated alveolar development and angiogenesis are critical for lung maturation as a gas exchange organ [25-27]. Inhibition of Flk-1 by specific inhibitor SU5416 resulted in decreased alveolarization in developing lung [25,27], emphysema [26], and severe hypoxic pulmonary hypertension in adult [28], indicating the fundamental roles of Flk-1 in lung development and maintenance of homeostasis in the pulmonary circulation. Although VEGF receptors have been characterized extensively at the level of expression, high affinity VEGF binding, phosphorylation, and other signal transduction properties, very little is known about factors which regulate its expression in endothelial cells [2,24]. An understanding of the mechanisms that underlie the transcriptional regulation of the Flk-1/KDR gene might provide important information about the molecular basis of endothelial cell differentiation, vascular development, and further assist our understanding in pulmonary angiogenesis. In the present study, we found that HIMF enhances Flk-1 expression in mouse lung tissues and endothelial cell line by activation of the PI-3K/Akt-NF-κB signaling pathway. In addition, our recent studies indicated that VEGF expression in lung epithelial cells can be induced by HIMF via the same signaling pathway [17], suggesting that additional transcription factors are involved in HIMF-mediated cell type-specific modulation of VEGF and its receptor Flk-1. Furthermore, HIMF, as it has dual function in upregulation of VEGF in epithelial cells and its receptor in endothelial cells, may serve as a coordinator in the control of pulmonary development and maturation, which certainly warrants further investigation.

Both mouse (Flk-1) and human (KDR) genes reveal a class II promoter structure, characterized by the absence of a TATA box and by the presence of several conserved *cis*-regulatory elements, including Sp1-, AP-2-, NF-κB-, and GATA-binding sites [22,29]. The upstream NF-κB site has been demonstrated to be the important one in mediating basal expression of the Flk-1/KDR promoter [30]. In addition, an overlapping palindromic GATA sequence plays a role in mediating constitutive promoter activity [30]. It has been previously shown that TNF-α activates NF-κB function to enhance human KDR expression [11], while TGF-β inhibits Flk-1/KDR expression through a mechanism that involves reduced binding of GATA-2 to a palindromic GATA site in the 5'-UTR [30]. These findings indicate that the binding of specific sets of transcription factors to the promoter region is necessary to modulate the expression of Flk-1 in response to different stimuli. In the current study, we found that HIMF protein upregulated Flk-1 expression by enhancing the Flk-1 promoter activity, rather than stabilizing Flk-1 mRNA posttranscriptionally. Moreover, the NF-κB activity was induced by HIMF administration or HIMF overexpression. Impairing NF-κB binding to the Flk-1 promoter via site-directed mutation or deletion abolishes HIMF-induced Flk-1 transcription, demonstrating a critical role of NF-κB in HIMF-mediated Flk-1 upregulation. In addition, we also found that deletion of binding sites for transcription factors E-box, Sp-1, and AP-2 partially attenuated HIMF-induced Flk-1 transcription, indicating that these transcription factors in the Flk-1 promoter also participate in HIMF-induced Flk-1 upregulation. The activation and interaction of these transcription factors and their correlation with NF-κB activity warrant our further study in the future.

The stimulating effects of HIMF on Flk-1 upregulation in SVEC 4–10 cells can only maintain for 24 hours. The dramatic decrease of NF-κB activity at 48 hour time point might be a result of HIMF degradation because we only administered the HIMF protein at the beginning of the experiment. These effects parallel with the activation of IKK and increased PI-3K activities as we showed that blocking IKK or PI-3K abolished HIMF-induced NF-κB activity and decreased Flk-1 mRNA production. The quick degradation or lost activity of HIMF further indicates that HIMF is a cytokine-like molecule and an early response gene to hypoxia, inflammation or other stress related stimuli [13,14].

NF-κB is composed of heterodimers of DNA-binding subunits (p50 and p52) and subunits with transcriptional activity (RelA, RelB, or c-Rel) [31]. In unstimulated cells, binary complexes of these subunits are restricted to the cytoplasm by interaction with members of a family of inhibitory proteins, inhibitors of κB (IκBs) [32]. In response to extracellular stimuli, phosphorylation of IκBα on serines 32 and 36 and of IκBβ on serines 19 and 23 facilitate their ubiquitination on neighboring lysine residues, thereby targeting these proteins for rapid degradation by the proteosome [32]. Dissociation from IκBs unmasks the nuclear localization sequence of NF-κB, permitting it to move into the nucleus, bind the promoters of target genes, and subsequently alter gene expression [33]. Although NF-κB can be activated by different stimuli, a high molecular weight IκB kinase (IKK) complex, termed IKK signalsome, serves as the key point that converges diverse upstream signals [23]. Activated IKK complexes phosphorylate IκB proteins, promoting their dissociation from NF-κB [23]. In the present study, we found that HIMF administration induced phosphorylation of IKK and IκBα. Moreover, transfection of the dominant-negative mutants of IKKα and IKKβ, and an IκBα super-repressor abolished HIMF-induced NF-κB activation. These data support the notion that HIMF activates NF-κB through phosphorylation of IKK and IκBα.

Phosphatidylinositol 3-kinase (PI-3K) is a heterodimer of an 85-kDa (p85) adaptor subunit and a 100-kDa (p110) catalytic subunit [34]. PI-3K activation has been linked to a number of biological processes such as cell survival, membrane trafficking, and insulin-stimulated glucose transport [35]. The serine-threonine protein kinase Akt is a downstream target of PI-3K-generated signals. A number of different growth factors have been shown to rapidly activate Akt via PI-3K signaling, such as platelet derived growth factor, epidermal growth factor, bFGF, insulin, and insulin-like growth factor 1 [36]. Akt may affect NF-κB through multiple mechanisms. It has been demonstrated previously that TNF-α activates Akt, which phosphorylates and activates IKKα, thus promoting NF-κB function [37]. Interleukin-1 can also increase the transactivation potential of the RelA subunit of NF-κB through a mechanism in which Akt has been implicated [38]. Our results demonstrated that HIMF induced Akt phosphorylation in SVEC 4–10 cells. The time-course of Akt phosphorylation is compatible with that of NF-κB activation in HIMF stimulated cells. Pretreatment of cells with LY294002, a PI-3K specific inhibitor, attenuated HIMF-induced Akt phosphorylation. Further, transfection of Δp85 blocked HIMF-induced phosphorylation of the IKK and IκBα, NF-κB activation, and thus prevented upregulation of Flk-1. These results provided strong evidence of HIMF induced cell signaling in endothelial cells via PI-3K/Akt, which cross talks with NF-κB, in the mediation of Flk-1 upregulation.

In summary, the current studies indicated that HIMF enhances Flk-1 expression in mouse lung tissues and endothelial cells in a PI-3K/Akt-NF-κB signaling pathway-dependent manner, which at least in part, elucidated the molecular mechanisms of transcriptional regulation of the Flk-1/KDR gene and contributed to our better understanding of the functions of HIMF in pulmonary angiogenesis and maintenance of pulmonary vascular homeostasis.
